# How Does Gelseminum Destroy Life?

**Published:** 1869-01-01

**Authors:** Z. C. McElroy

**Affiliations:** Zanesville, Ohio, President Muskengum County Medical Society


### HOW DOES GELSEMINUM DESTROY LIFE?—ANTIDOTES AND REMEDIAL MANAGEMENT.

By Z. C. McElroy, M.D., Zanesville, Ohio, President Muskengum County Medical Society.

The case of poisoning by the *Fl. Ex. Gelseminum*, reported by Dr. W. F. Hané, in the Journal for December 1st, 1868, must be the writer's apology for endeavoring to communicate some knowledge on the subject of poisoning of a general character, applicable to Dr. H.'s case, as well as to all other cases of poisoning.

As physiology and pathology advance in their course, to their ultimate positions of exact sciences, definitions must necessarily be narrowed down to embrace but single points. The term poisoning is exceedingly loose, and is to be taken in no sense as scientific. It is strictly popular. A few propositions may serve to place the whole subject definitely before the mind.

*1st.* The forces, powers, or dynamics of the human body are derived from the oxidation, waste, or destructive metamorphosis of its own nerve and other tissues; with, possibly, the oxidation of some articles consumed as food, without passing into organized tissue. The word *possibly* is used understandingly, for only the first part of the proposition is definitely known. The second part may *possibly* be true, but it awaits demonstration.

*2nd.* Articles classed as poisons in the professional, as well as in the popular mind, have two modes, at least, of producing death, viz.: 1st. By arrresting destructive metamorphosis in the nerve masses supplying the dynamics of the brain, heart, or lungs. 2nd. Or directly the reverse of this, by promoting destructive metamorphosis in the same nerve masses, and furnishing excess of dynamic force to the same organs.

*3rd.* Fluids, in or out of the living body, obey the ordinary laws of gravitation, when not controlled by superior forces or dynamics. Applying these principles to explain the mode of death by *Gelseminum*, it will be seen, that, taking the symptoms as detailed by Dr. H. (and they are correct), that death was due to paralysis of the heart and lungs. This paralysis is due to an arrest of destructive metamorphosis in the cerebellum, and upper portion of the spinal cord; though, very probably, the paralysis was general, but death was due to arrest of oxidation in these particular parts. *Gelseminum*, then, produces death by suspending, or by furnishing conditions suspending molecular metamorphosis or waste in the nerve masses, supplying the dynamics of the circulation and respiration.

*Gelseminum* is but one of a class of which *Opium*, *Belladonna*, *Chloroform*, *Aconite*, etc., are examples of others. *Strychnia* is an example of the other class, producing death in a mode precisely opposite to *Gelseminum*. It furnishes conditions for increased destructive metamorphosis in the same nerve masses supplying the dynamics of the circulation and respiration; the excessive force liberated, interrupting the alternate contractions and expansions of the lungs and heart, producing permanent contraction of the heart, and the muscles of respiration. As with *Gelseminum*, the paralysis was general, so with *Strychnia*, the contractions of the muscles are general, every thing rigid, and death due to excessive dynamics.

In one or the other of these two ways all poisons act which do not at once destroy tissue, as *Acids*, *Caustic Alkalies*, etc. In the not distant future these distinctions will be recognized in text-books.

The mode by which *Gelseminum*, and other agents of its class, produce death, being thus definitely determined, the indications for treatment are about as follows:

1*st.* If seen sufficiently early, prompt evacuation of the stomach should be obtained by emetics, or by the stomach-pump. If, after the evacuation of the stomach, the symptoms continue to deepen, other measures must be relied on to carry the patient through the crisis. They would be:

2*nd.* Place patient on table, or board, with supports in the middle and at both ends. As the paralysis increases, the circulation comes under the control of the ordinary laws of gravitation, and settles to the most dependent positions of its circuit in the dying body. Now, to make this gravity an aid in prolonging life, alternately depress the head and feet of the patient. As the head is inclined to an angle of 45°, the blood fills the brain, and dependent portion of the body, at the expense of, what for the moment is, the upper, including the lungs. Reverse the position, placing the head at an angle of 45° elevation, the circulation will flow to the dependent parts of the body, which are for the moment again the abdomen, and legs, though the lungs will receive their share in this position, as they lose it when the head is inclined. That is what may be called the conversion or correlation of gravity into organic force. As life may be sustained with ten to twenty pulsations of the heart, per minute, this alternate elevation and depression of the head should take place every three to five seconds. Brisk friction to and from the brain would aid considerably.

*3rd.* Artificial respiration. Inflating the lungs by Marshall Hall's method, or by any other way, so that air charged with its due proportion of oxygen may be forced in, and not the breath of another person, charged with carbonic acid. As the artificial respiration conveys the life-giving, as well as the molecularly destructive oxygen into the lungs, in such way as to render the oxygenation and decarbonization of the blood possible, destructive metamorphosis may be kept proceeding at a reduced rate, and some power or dynamic force liberated to help carry on the respiration and circulation, and the patient out of a position otherwise hopeless. The lungs should be inflated four to eight times per minute.

*4th.* As *Strychnia* is an energetic agent in promoting destructive metamorphosis in the nerve masses, in which *Gelseminum* suspends it, it may be profitably used by hypodermic injection, near the medulla oblongata, in doses from $\frac{1}{20}$ to $\frac{1}{100}$ of a grain, according to the extent of the existing paralysis. As a further aid, magnetism or electricity may be passed through the spinal cord. As the condition depends on diminished destructive metamorphosis, and as low temperatures have the same general tendencies, the patient should be surrounded by as high a temperature as the assistants can comfortably bear, if in a room with registers, fire places, or stoves. If otherwise, by artificial heat in any other way, applied immediately to the person of the patient.

*5th.* If the patient can swallow water, with salines, such as *Bi Carb. Soda* or *Potash*, *Chlorate Potassa*, *Carbonate of Ammonia*, or *Rochelle Salts*, in solution, may be given as they facilitate or promote destructive metamorphosis and elimination. If the efforts to sustain life are protracted, the bladder should be emptied by catheter.

Brandy and *Opium* are agents restraining waste or destructive metamorphosis, and are, therefore, not indicated in the treatment of the paralysis, or suspended destructive meta morphosis, from *Gelseminum*, through appropriate in poisoning, or excessive destructive metamorphosis, induced by *Strychnia*, in over-doses.

Such are the principal means for combatting poisoning by *Gelseminum*, and all other agents of its class. It is not at all probable that any direct antidote will ever be found acting in the same sense as alkalies act in neutralizing surplus acid in the stomach, to the effects of *Gelseminum*.

The apparent antagonisms, or antidotal effects of *Opium* and *Belladonna*, to the effects of each other, are, to the writer, for the present, an unexplained riddle. Certain it is, however, that combinations of *Opium* and *Belladonna*, in doses of ten drops of a solution of eight grains of *Morphia* to one grain of *Atropia*, in one ounce of water, by hypodermic syringe, expands the pupil widely, and induces the effects of both agents in the system at the same time, as has been verified by the experience of the writer. The reputed antagonisms of *Opium* and *Belladonna*, though apparently illustrated in the same number of the Journal, by Dr. Newman, must for the present, to me, remain a mystery.

